# Supplementary material for: Prevalence and factors associated with long COVID and mental health status among recovered COVID-19 patients in southern Thailand
Source: PLoS One. 2023 Jul 31;18(7):e0289382. doi: 10.1371/journal.pone.0289382 (PMC10389739; doi:10.1371/journal.pone.0289382)
Supplement: S1 Checklist — (PDF) [file pone.0289382.s002.pdf]

STROBE Statement—checklist of items that should be included in reports of observational studies

	Item No.	Recommendation	Page No.	Relevant text from manuscript
**Title and abstract**	1	(*a*) Indicate the study's design with a commonly used term in the title or the abstract	3	This study was conducted using a cross-sectional study to investigate the prevalence and potential risk factors of long COVID and mental health disorders among individuals who had recovered from COVID-19 in Nakhon Si Thammarat province, southern Thailand.
		(*b*) Provide in the abstract an informative and balanced summary of what was done and what was found	3	A total of 939 participants were randomly selected for the study. The Depression, Anxiety, and Stress Scale-21 (DASS-21) investigated mental health disorders among COVID-19 participants. And a checklist comprised of thirteen common symptoms was used to identify the long COVID condition. Logistic regression models were used to investigate the risk factors associated with mental health disorders and long COVID symptoms among participants.<br><br>Among the 939 participants, 104 (11.1%) reported experiencing depression, 179 (19.1%) anxiety, and 42 (4.8%) stress. A total of 745 participants (79.3%) reported experiencing at least one symptom of long COVID. Fatigue (72.9%, SE ± 0.02), cough (66.0%, SE ± 0.02), and muscle pain (54.1%, SE ± 0.02) were the most frequently reported symptoms. All long COVID symptoms were significantly associated with mental health disorders, with shortness of breath, fatigue, and chest tightness being the most significant risk factors for mental health disorders among COVID-19 patients. Additionally, female patients (OR = 1.89), medical history (OR = 1.92), and monthly income lower than 5,000 Thai baht (OR = 2.09) were associated with developing long COVID symptoms and mental health disorders (all $p < 0.01$).
**Introduction**				
Background/rationale	2	Explain the scientific background and rationale for the investigation being reported	5	Mental health disorders have emerged as a pressing public health concern for individuals who have recovered from COVID-19. Previous studies suggested a potential link between the psychological symptoms of COVID-19 patients and the onset of long COVID. This highlights the importance of identifying the risk factors associated with mental health disorders and post-COVID-19 conditions.

Objectives	3	State specific objectives, including any prespecified hypotheses	6	The study aims to:<br>(1) examine the prevalence of long COVID and mental health disorders among Thai adults who have recovered from COVID-19,<br>(2) identify the association between mental health issues such as depression, anxiety and stress and long COVID symptoms among COVID-19 participants,<br>(3) investigate the risk factors associated with the correlation between mental health outcomes and the onset of long COVID in adult patients who have previously contracted COVID-19
**Methods**				
Study design	4	Present key elements of study design early in the paper	6-10	The critical elements of a study design early in a paper typically include the following information:<br>- Study type: observational study (a cross-sectional study)<br>- Participants: Thai adults who have recovered from COVID-19, were over 18 years old, had no mental health disorders diagnosed in the past, and got COVID-19 infection between January and May 2022.<br>- Study area: in a community setting in subdistricts of Sichon district, Nakhon Si Thammarat Province, southern Thailand.<br>- Data collection: a survey using a structured questionnaire and medical records.<br>- Variables: socio-demographic information, body mass index (BMI), medical history, checklist (Yes/No) of thirteen common long COVID symptoms; and Depression, Anxiety and Stress scores using DASS-21.<br>- Data analyses: Prevalence of long COVID, mental health disorders and characteristics of study participants were measured using descriptive analyses. Risk factors analyses were applied using logistic regression models.
Setting	5	Describe the setting, locations, and relevant dates, including periods of recruitment, exposure, follow-up, and data collection	7, 9	The study was conducted in the community setting across nine subdistricts of Sichon district, located in Nakhon Si Thammarat Province, southern Thailand. Only individuals who recovered from COVID-19 between January and May 2022 were selected to participate in our study. To ensure sufficient time had elapsed for long COVID conditions, the study was conducted in November 2022, which corresponds to three months after the patients contracted COVID-19 and at least two months of experiencing symptoms indicative of long COVID. Fourteen village health volunteers and study staff members were assigned to the

				nine subdistricts to collect data for the study.
Participants	6	(*a*) *Cohort study*—Give the eligibility criteria, and the sources and methods of selection of participants. Describe methods of follow-up<br>*Case-control study*—Give the eligibility criteria, and the sources and methods of case ascertainment and control selection. Give the rationale for the choice of cases and controls<br>*Cross-sectional study*—Give the eligibility criteria, and the sources and methods of selection of participants<br>(*b*) *Cohort study*—For matched studies, give matching criteria and number of exposed and unexposed<br>*Case-control study*—For matched studies, give matching criteria and the number of controls per case	7	Eligible participants were selected based on a list provided by databases from two secondary care hospitals and three field hospitals. Only individuals over 18 years old with no mental health disorder before contracting COVID-19 were included in the study. Additionally, only individuals who had recovered from COVID-19 between January and May of 2022 were deemed eligible to participate in our research. We conducted this study in November 2022 to ensure adequate time had passed for the incidence of long COVID conditions (i.e., three months after the patients contracted COVID-19 and at least two months experiencing symptoms characterized as long COVID). The sample size calculation was based on the population size (N) of 9,396 eligible participants.
Variables	7	Clearly define all outcomes, exposures, predictors, potential confounders, and effect modifiers. Give diagnostic criteria, if applicable	10	Logistic regression was utilized to identify risk factors associated with mental health status and long COVID among COVID-19 patients. In order to perform logistic regression analysis, the two binary variables of mental health status and long COVID were merged into a new variable with four levels. A binary outcome variable was created for logistic regression modelling, where observations with experience of both mental health status and long COVID were categorized as “1”, while the three other levels of the new variable were considered “0”. Explanatory variables (predictors) included: (1) gender, (2) age, (3) marital status, (4) education, (5) occupation, (6) monthly income, (7) BMI, and (8) medical history<br>Univariable models were initially screened for all explanatory variables, and those with $P < 0.20$ were selected as candidates for the final model. The multivariable model consisted of variables with $P < 0.05$ and was used to identify significant risk factors associated with mental health status and long COVID in COVID-19 patients. Multivariable models are used

				in statistical analyses to control for potential confounders
Data sources/ measurement	8*	For each variable of interest, give sources of data and details of methods of assessment (measurement). Describe comparability of assessment methods if there is more than one group	7,8	The study involved administering a structured questionnaire to the participants, which consisted of three sections.<br>- The first section aimed to collect socio-demographic information: age, gender, education level, marital status, occupation, and monthly income. Additional data were also collected on height, weight, underlying diseases of all participants. Body mass index (BMI) was calculated using the weight to-height squared (kg/m^2) ratio, and individuals were categorized as underweight, normal weight, overweight at risk, or obese, based on their BMI values (<18.5, 18.5–22.9, 23.0–24.9, and ≥ 25.0, respectively).<br>- The second section of the questionnaire consisted of 13 commonly reported symptoms associated with long COVID included fatigue, shortness of breath, chest tightness, palpitations, cough, amnesia, insomnia, joint pain, muscle pain, asthenia, significant hair loss, headache, and dizziness. The participants were asked "Yes/No" questions to indicate whether they had experienced these symptoms for a period of two months after three months infected with SARS-CoV-2. Those who reported experiencing at least one symptom were classified as having long COVID.<br>- The third section involved the use of the 21-item Depression Anxiety and Stress Scale (DASS-21) developed by Lovibond and Lovibond (1995). The DASS-21 was used to assess the emotional states of the participants in relation to the three mental health disorders: depression (7 items), anxiety (7 items), and stress (7 items). Each term was rated on a 4-point scale, ranging from "did not apply to me at all" (0 points), "applied to me some degree or some of the time" (1 point), "applied to me to a considerable degree or a good part of the time" (2 points), and "applied to me very much or most of the time" (3 points). Final scores for each mental health disorder were calculated by adding relevant items and multiplying them by two. The severity levels were categorized as follows: depression (normal: 0 - 9; mild: 10 - 13; moderate: 14 - 20; severe: 21 - 27; extremely severe: ≥ 28); anxiety (normal: 0 - 9; mild: 8 - 9; moderate: 10 - 14; severe: 15 - 19; extremely severe: ≥ 20), and stress (normal: 0 - 14; mild: 15 - 18; moderate: 19 - 25; severe: 26 - 33; extremely severe: ≥ 34). Participants were then categorized based on their scores as "normal", "mild", "moderate", "severe", and "extreme severe" for each disorder. Participants who scored in the "mild" to "extremely severe" range were considered to have

				mental health disorders.
Bias	9	Describe any efforts to address potential sources of bias	7, 9, 10	Several sources of bias can affect observational studies. To address these potential biases, we took the following measures in our research:<br>- *Selection bias:* We randomly selected study participants from a list of eligible individuals to minimize selection bias.<br>- *Information bias*: Our effort to minimize the information bias was as follows: First, we included only participants with no mental health disorders before contracting COVID-19. Second, we used a standardized and structured questionnaire, evaluated for content validity, to help participants accurately remember and report their experiences. Third, we collected data in November 2022, the proper time after participants contracted COVID-19 (between January and May 2022). Shorter recall periods are recommended for data collection to reduce the chances of recall bias.<br>- *Confounders*: We used multivariable analyses to reduce our study's potential for confounding biases.
Study size	10	Explain how the study size was arrived at	7	The target sample size of approximately 900-950 representative participants was determined using a web-based sample size calculation tool (http://www.winepi.net). This calculation was based on a reported prevalence of 57% of COVID-19 survivors experiencing long COVID, a population size (N) of 9,396 from the hospital databases, a margin of error (d) of around 3%, and a confidence interval of 95%.
Quantitative variables	11	Explain how quantitative variables were handled in the analyses. If applicable, describe which groupings were chosen and why	9,10	- For socio-demographic data: (age, gender, education level, marital status, occupation, monthly income, BMI): quantitative variables were grouped into categories and shown using percentage (%) for each type.<br>- Descriptive statistics were applied to calculate (prevalence, standard error) to summarize the prevalence of mental health disorders and long COVID symptoms among participants.<br>- Apart from calculating the frequency of each long COVID symptom, we calculated a standardized score for each COVID symptom in case the participant experienced more than one symptom. This score was calculated by multiplying the rank of each symptom by its share in each participant's observations, which totalled 100%. For example, if a participant

				mentioned 3 out of 13 symptoms, such as fatigue, shortness of breath, and chest tightness, each would receive a standardized score of 33.3%.
Statistical methods	12	(*a*) Describe all statistical methods, including those used to control for confounding	9, 10	- The association between socio-demographic factors and long COVID among participants was examined using the Chi-squared test and Fisher's Exact test.<br>- In addition, we hypothesized that the mental health conditions of COVID-19 patients were likely to be associated with the symptoms of long COVID. To test this hypothesis, we used the odds ratio (OR) to examine the association between depression, anxiety and stress and each symptom of long COVID.<br>- Logistic regression was utilized to identify risk factors associated with mental health status and long COVID among COVID-19 patients. In order to perform logistic regression analysis, the two binary variables of mental health status and long COVID were merged into a new variable with four levels. A binary outcome variable was created for logistic regression modelling, where observations with experience of both mental health status and long COVID were categorized as "1", while the three other levels of the new variable were considered "0". Explanatory variables (predictors) included: (1) gender, (2) age, (3) marital status, (4) education, (5) occupation, (6) monthly income, (7) BMI, and (8) medical history. Univariable models were initially screened for all explanatory variables, and those with $P < 0.20$ were selected as candidates for the final model. The multivariable model consisted of variables with $P < 0.05$ and was used to identify significant risk factors associated with mental health status and long COVID in COVID-19 patients. Multivariable models are used in statistical analyses to control for potential confounders
		(*b*) Describe any methods used to examine subgroups and interactions	10	Interactions between all pairs of explanatory variables were examined to determine any potential confounding effects between the explanatory variables.
		(*c*) Explain how missing data were addressed	7, Support-ting Infor-mation	Using a structured questionnaire, we ensured no data were missed during the data collection (please refer to the raw data attached in the Supporting Information). However, in the event of any missing data and considering the potential impact of such missing data, various approaches (i.e., complete case analysis, mean/mode imputation, or multiple imputations) may be applied.
		(*d*) *Cohort study*—If applicable, explain how loss to follow-up was addressed	7	The sample size calculation was based on the population size (N) of 9,396 eligible participants (as a finite population).

		Case-control study—If applicable, explain how matching of cases and controls was addressed<br>*Cross-sectional study*—If applicable, describe analytical methods taking account of sampling strategy	A web-based sample size calculation tool determined the target sample size of approximately 900-950 representative participants (http://www.winepi.net). This calculation was based on a reported prevalence of 57% of COVID-19 survivors experiencing long COVID, a population size (N) of 9,396 from the hospital databases, a margin of error (d) of around 3%, and a confidence interval of 95%.
		(*e*) Describe any sensitivity analyses	Not applicable
**Results**			
Participants	13*	(a) Report numbers of individuals at each stage of study – eg. numbers potentially eligible, examined for eligibility, confirmed eligible, included in the study, completing follow-up, and analysed	We selected individuals from a database of two secondary care hospitals and three field hospitals containing information on 10,336 participants who had recovered from COVID-19 between January and May 2022. Only individuals over 18 years old and with no history of mental health disorders before contracting COVID-19 were included in the study. Ultimately, 9,396 eligible participants were deemed suitable to participate in our research.<br>To determine the target sample size, we used a web-based sample size calculation tool (http://www.winepi.net). Based on a reported prevalence of 57% of COVID-19 survivors experiencing long COVID, a population size (N) of 9,396 from the hospital databases, a margin of error (d) of approximately 3%, and a confidence interval of 95%, a representative sample size of roughly 900-950 participants was determined.<br>Proportional allocation using stratified sampling was employed to select participants from each subdistrict randomly. As a result, 939 participants were included in this study.
		(b) Give reasons for non-participation at each stage	In the case of participants being absent from their houses during data collection or refusing to participate in the study from the randomly selected list, a solution was implemented to address these situations. An additional participant was randomly recruited from a reserved list, ensuring adequate sample size was maintained for analysis.

		(c) Consider use of a flow diagram		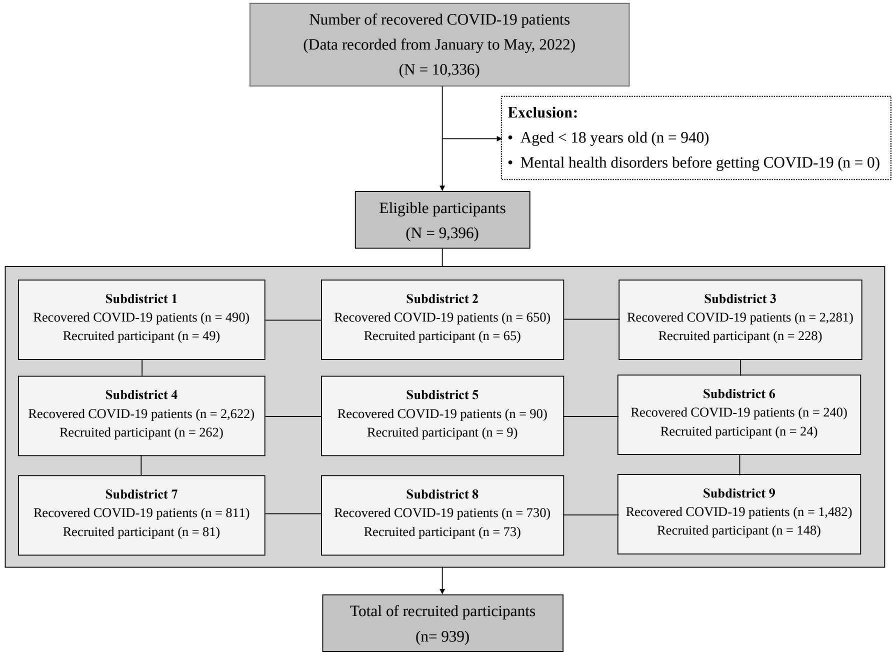
Descriptive data	14*	(a) Give characteristics of study participants (eg demographic, clinical, social) and information on exposures and potential confounders	11	A total of 939 participants were included in this study, of whom 745 (79.3%) reported experiencing at least one symptom lasting for more than two months, indicating prolonged COVID-19 symptoms, while 194 participants (20.7%) reported no history of long COVID symptoms. The majority of respondents were female (77.4%), and a significant gender difference was observed in the development of long COVID ($\chi 2$ test, $p < 0.001$). Most participants were younger than 60 (84.7%), and no significant association was found between long COVID and age groups either under or over 60 years old ($\chi 2$ test, $p = 0.867$). Approximately 69.1% of participants were currently married, and a significant difference was observed in the development of long COVID based on marital status ($\chi 2$ test, $p = 0.021$). The study found that around 80% of participants reported a high school education or lower, with self-employment being the most common occupation. However, no significant association was found between long COVID and education or occupation ($\chi 2$ test and Fisher's exact test, all $p \geq 0.168$). A majority (90%) of study participants earned a monthly

				income of less than 15,000 Thai baht (~ 450 USD), and a significant association was found between long COVID symptoms and monthly income levels ($\chi2$ test, $p = 0.009$). Sixty-five percent of participants were overweight (BMI > 23.0), and 44% were obese. The study found significant associations between increased BMI levels and long COVID symptoms. While no significant associations were found between long COVID and most historical diseases reported by participants, such as hypertension, diabetes, cardiovascular disease, or allergies (all $p \geq 0.154$), a significant association was observed for dyslipidemia status among participants ($\chi2$ test, $p < 0.001$).
		(b) Indicate number of participants with missing data for each variable of interest	Support-ting Infor-mation	We ensured no data were missed during the data collection (please refer to the raw data attached in the Supporting Information)
		(c) *Cohort study*—Summarise follow-up time (eg, average and total amount)		
Outcome data	15*	*Cohort study*—Report numbers of outcome events or summary measures over time<br>*Case-control study*—Report numbers in each exposure category, or summary measures of exposure		
		Cross-sectional study—Report numbers of outcome events or summary measures	13,14	*The characteristics of long COVID symptoms among participants*<br>Out of the 939 participants, 745 (79.3%) reported experiencing at least one symptom of long COVID. The median number of symptoms reported was 4, with an interquartile range (IQR) of one to seven. Among COVID-19 patients with long COVID, more than half of the participants experienced fatigue (72.9%, SE ± 0.02), cough (66.0%, SE ± 0.02), and muscle pain (54.1%, SE ± 0.02), with median standardized scores of 10.0 [IQR 0-16.7], 8.3 [IQR - 16.7], and 0.0 [IQR 0-12.5], respectively. The remaining symptoms of long COVID were less frequently reported, with median standardized scores of zero. The frequency of these symptoms was as follows: insomnia (49.4%, SE ± 0.02), headache (48.7%, SE ± 0.02), joint pain (45.0%, SE ± 0.02), shortness of breath (43.5%, SE ± 0.02), dizziness (41.7%, SE ± 0.02), amnesia (41.2%, SE ± 0.02), hair loss (29.7%, SE ± 0.02), palpitations (24.8%, SE ± 0.02), chest tightness (15.3%, SE ± 0.01), and asthenia (12.8%, SE ± 0.01).

				Depression, Anxiety and Stress among recovered COVID-19 participants<br>Out of 939 participants, 104 (11.1%) were found to have depression, with severity ranging from mild (35 participants) to extremely severe (4 participants). For anxiety, 179 (19.1%) of participants were diagnosed with varying levels of severity, including mild (42 participants), moderate (111 participants), severe (15 participants), and extremely severe (11 participants). Stress was identified in only 42 participants (4.8%), mostly at a mild level (25 participants), followed by moderate (11 participants), severe (5 participants), and extremely severe (1 participant). Among those who had recovered from COVID-19, 33 (3.5%) were diagnosed with all three mental health disorders of depression, anxiety, and stress. There were 70 (7.5%) participants had two out of the three mental health conditions, and 189 (20.1%) had at least one of the three mental health issues.
Main results	16	(*a*) Give unadjusted estimates and, if applicable, confounder-adjusted estimates and their precision (eg, 95% confidence interval). Make clear which confounders were adjusted for and why they were included	17	The adjusted estimates of were represented in the univariable models in Table 3 with their precision (95% CI) and P-value.
		(*b*) Report category boundaries when continuous variables were categorized	Table 1	Only one continuous variable, “age”, was categorized into two subsets (19-59 vs ≥ 60). We hypothesized the advanced age might be a risk factor for long COVID and mental health disorders. However, there was no significant association between the age of participants and long COVID and mental health disorders in our study.
		(*c*) If relevant, consider translating estimates of relative risk into absolute risk for a meaningful time period		Not applicable
Other analyses	17	Report other analyses done — eg analyses of subgroups and interactions, and sensitivity analyses	16	No significant interactions were found between potential explanatory variables in multivariable model.
**Discussion**				

Key results	18	Summarise key results with reference to study objectives	18	Our study has revealed that depression, anxiety, and stress are not prevalent among COVID-19 adults after being discharged from the hospital. However, a high prevalence of long COVID symptoms was observed, with fatigue, cough and muscle pain being the most common. The study also identifies shortness of breath, fatigue, and chest tightness as the highest risk factors for mental health disorders among COVID-19 patients who experience such issues. Finally, the study finds that female patients, medical history of COVID-19 patients, and low income are associated with the development of long COVID symptoms and mental health disorders among the study participants.
Limitations	19	Discuss limitations of the study, taking into account sources of potential bias or imprecision. Discuss both direction and magnitude of any potential bias	21	Our study has some limitations.<br>First, as mental health problems can have long-lasting effects, cross-sectional studies may not identify the long-term impacts of mental health issues and long COVID symptoms among COVID-19 patients. Thus, follow-up studies using cohort investigations are recommended.<br>Additionally, our research was conducted only in rural communities in southern Thailand, and therefore, the prevalence and factors associated with long COVID symptoms and mental health disorders in other areas may differ (i.e., urban and suburban areas). Although our findings may be generalizable to rural areas, further research is needed to comprehensively understand the prevalence of factors associated with long COVID and mental health issues among COVID-19 patients in various regions of Thailand.
Interpretation	20	Give a cautious overall interpretation of results considering objectives, limitations, multiplicity of analyses, results from similar studies, and other relevant evidence	18-21	The findings of our study need to be interpreted cautiously, considering several factors.<br>Firstly, it is essential to acknowledge that the prevalence of mental health disorders among COVID-19 patients in our study was lower compared to previous research conducted in Thailand. This discrepancy may be attributed to differences in participant selection and the timing of the survey. Our study focused on participants from field hospitals, while previous studies included patients from hospitals or high-risk districts. Additionally, our study was conducted after lifting strict COVID-19 restrictions, which may have improved the overall well-being of rural communities. Another contributing factor could be the increased attention and availability of mental health resources and services in the Thai healthcare system, which may have effectively reduced mental health disorders among COVID-19 patients.<br>However, it is essential not to overlook the long-term effects of mental health issues and the

need for ongoing monitoring and research in this area. The combination of symptoms experienced by COVID-19 patients with long COVID can vary widely, with fatigue, shortness of breath, chest pain, and other symptoms being commonly reported. These symptoms can persist for extended periods, and their severity can differ among individuals. The underlying mechanisms causing the development of long COVID symptoms are not yet fully understood and require further investigation.

Our study also found that long COVID symptoms are significant risk factors for developing mental health disorders among COVID-19 patients. This finding aligns with recent studies linking long COVID symptoms, such as fatigue and respiratory issues, to higher risks of depression, anxiety, and stress disorders. The distress and disruption to daily life caused by long COVID symptoms and the uncertainty surrounding recovery may contribute to developing mental health problems. The neurological effects of long COVID could also play a role in the manifestation of mental health issues. However, additional research is needed to understand better the relationship between long COVID symptoms and mental health disorders.

Furthermore, our study identified that female COVID-19 patients and those with a history of COVID-19 infection are more likely to experience long COVID and mental health problems. This finding is consistent with previous research indicating that females are at higher risk of long COVID and that individuals with a prior history of mental illnesses face increased risks. Biological factors, such as immune responses and social and cultural factors, including caregiving responsibilities and gender roles, may contribute to these associations. In addition, our research highlighted that individuals with lower incomes are at a heightened risk of developing mental health issues and long COVID. Financial stress, limited access to healthcare services, and social and economic factors could all contribute to these disparities among COVID-19 patients with lower incomes.

The findings support the need for continued monitoring, research, and the implementation of appropriate interventions to address the mental health challenges faced by COVID-19 patients, particularly those with long COVID symptoms.

Generalisability	21	Discuss the generalisability (external validity) of the study results	5-21	*Population Characteristics***:** The study focused on Thai adults who had recovered from COVID-19 in Nakhon Si Thammarat province, southern Thailand. The findings may apply more to individuals with similar demographic and cultural backgrounds. However, caution should be exercised when generalizing the results to other populations since factors such as healthcare systems, socioeconomic conditions, and cultural norms can vary across regions and countries.<br><br>*Study Design***:** The study applied a cross-sectional design. As mental health problems can have long-lasting effects, cross-sectional studies may not identify the long-term impacts of mental health issues and long COVID symptoms among COVID-19 patients. Thus, follow-up studies using cohort investigations are recommended.<br><br>*Sampling***:** While the study employed random selection to recruit participants from a specific province, its generalizability beyond the local area may be limited. Regional variations in demographics, healthcare access, and COVID-19 outcomes could affect the applicability of the findings to the broader Thai population or other countries. Furthermore, the study focused exclusively on rural communities in southern Thailand, which might lead to differences in the prevalence and factors associated with long COVID symptoms and mental health disorders in other regions. Although the findings may apply to rural areas, further research is necessary to understand the prevalence and factors related to long COVID and mental health issues among COVID-19 patients in various regions of Thailand.<br>*Measurement Tools***:** The study utilized the Depression, Anxiety, and Stress Scale-21 (DASS-21) to assess mental health disorders and a checklist of thirteen common symptoms to identify long COVID. These measurement tools have been widely used and validated, enhancing the reliability of the findings.<br>*Risk Factors and Associations***:** The study identified several risk factors associated with mental health disorders and long COVID symptoms, such as gender, medical history, and monthly income. While these findings provide insights into potential associations, the generalizability may depend on the similarity of risk factors across populations. Factors like healthcare access, cultural influences, and socioeconomic disparities may differ in other regions, which could affect the generalizability of the identified risk factors.<br>*Contextual Considerations***:** The study was conducted in a specific timeframe and geographical location. The generalizability of the findings may be influenced by the

			circumstances prevailing during the study period, such as the local COVID-19 situation, healthcare infrastructure, and public health measures. When applying the results to other settings or time periods, these contextual factors should be considered.<br>*Consistency with Previous Studies*: The results of this study are consistent with prior research that has highlighted the impact of COVID-19 on mental health and the persistence of long COVID symptoms.
**Other information**			
Funding	22	Give the source of funding and the role of the funders for the present study and, if applicable, for the original study on which the present article is based	This work was funded by the Excellent Center for Dengue and Community Public Health (EC for DACH), Walailak University (Ref. No number WU- COE-66-15), awarded to Charuai Suwanbamrung. The funder had no role in study design, data collection and analysis, and manuscript preparation. The funding was only for research purposes.

*Give information separately for cases and controls in case-control studies and, if applicable, for exposed and unexposed groups in cohort and cross-sectional studies.

Note: An Explanation and Elaboration article discusses each checklist item and gives methodological background and published examples of transparent reporting. The STROBE checklist is best used in conjunction with this article (freely available on the Web sites of PLoS Medicine at http://www.plosmedicine.org/, Annals of Internal Medicine at http://www.annals.org/, and Epidemiology at http://www.epidem.com/). Information on the STROBE Initiative is available at www.strobe-statement.org.
