# Supplementary material for: Prevalence and factors associated with long COVID and mental health status among recovered COVID-19 patients in southern Thailand
Source: PLoS One. 2023 Jul 31;18(7):e0289382. doi: 10.1371/journal.pone.0289382 (PMC10389739; doi:10.1371/journal.pone.0289382)
Supplement: Attachment — Submitted filename: PONE-D-23-13130.docx [file pone.0289382.s003.docx]

Manuscript No: PONE-D-23-13130

PLOS ONE

**Prevalence and Factors Associated with Long COVID and Mental Health Disorders among Recovered COVID-19 Patients in Southern Thailand**

Thank you for providing the opportunity to review this manuscript. The paper presents a potentially exciting study timely on the current problems. This article makes some important contributions in terms of identifying factors associated with long COVID and its association with mental health problems. The results of this study may have important policy implications in its field and may contribute to developing strategies for addressing the issues. However, reading through the paper, I suggest authors improve the manuscript with revisions in some sections to get published.

**Introduction**

The background section is inadequately described. Suggested to expand this section with clear problem statements, rationale and research question/s in a logical flow so that it helps the readers to understand the context and the reasons to conduct this study. Need to describe a bit about mental health situation in Thailand. For example, the information of mental health problem in Thailand before the pandemic. Also, stating the hypothesis or research question/s, and the rationale of the study in this section would be helpful for the readers to understand the study clearly.

L 92 - The study aims stated is not clear. It differs than previously stated aims in the abstract section. Suggested to make it consistent.

**Materials and Methods**

Reorganization of this section is required. Suggested moving the ethics paragraph at the end of this section. Please note that some Journal asks to move the ethics paragraph under the declaration section – follow the journal structure. However, it is recommended to start the method section with a paragraph describing the study setting/context followed by the study design, sample, and participant recruitment. In the study setting, you may want to provide information about the study area - geographical area, population, the effect of COVID-19 in the area (was the study area a highly affected area?), health facilities and services so on.

L 97 - The authors stated that the study protocol adhered to the principles outlined in the Declaration of Helsinki. What are they? Please specify the key principles.

L106 – 107 - This sentence is not related to design. This may go to context/setting. Study design could be Institution or community-based, cross-sectional design…...?

**Sample size and sampling:** L109 – 110 stated the approximate range of sample i.e. 900 – 950. What was the sample size calculated? Give the exact number.

In the result section L 203 stated the sample of 939. How many were approached for interviews and what was the non-response rate? Please describe it clearly.

**Data Collection:** L 165 – 170 - It confuses about the data collection process/procedure. Were the data collection tools and questionnaires self-administered by the participants using online survey form or VHVs collected data conducting face-to-face interviews? If it was self-administered, how BMI was measured? How the measurement accuracy was ensured?

**Data Analysis**: L 192 – stated that the explanatory variables with P < 0.20 in univariate analysis were selected for the final model. Why did you choose the variables with P value <0.20? Provide the reference for it.

**Results**

The characteristics of study participants and the result descriptions are mixed up that may create confusions to the readers. It is suggested to separate it starting with a paragraph of socio-demographic characteristics of the study participants followed by the result descriptions.

Also, a number of repetitions noted in presenting the results. For example, L 203 and L 229 – the same sentence/result is written twice.

Figure 1 and 2, both are poor and not readable. Could it be presented differently?

A table showing depression, Anxiety and Stress prevalence and severity could be helpful instead of figure.

Table 2: L 275. Asterisk (*) indication for p value is not properly done! P<0.01 must have indicated with ** (2 asterisk not 3).

**Discussion**

L 296 – 297 The study has revealed that depression, anxiety, and stress are not prevalent among COVID-19 adults after being discharged from the hospital. This finding or statement is confusing. It doesn’t seem true. Please reconfirm it.
